# Microstructural and Rheological Transitions in Bacterial Biofilms

**DOI:** 10.1002/advs.202207373

**Published:** 2023-07-31

**Authors:** Samuel G.V. Charlton, Amber N. Bible, Eleonora Secchi, Jennifer L. Morrell‐Falvey, Scott T. Retterer, Thomas P. Curtis, Jinju Chen, Saikat Jana

**Affiliations:** ^1^ Department of Civil, Environmental and Geomatic Engineering Institute of Environmental Engineering ETH Zurich Zurich 8049 Switzerland; ^2^ School of Engineering Newcastle University Newcastle Upon Tyne NE1 7RU UK; ^3^ Biosciences Division Oak Ridge National Laboratory Oak Ridge TN 37830 USA; ^4^ Center for Nanophase Material Sciences Oak Ridge National Laboratory Oak Ridge TN 37830 USA; ^5^ School of Engineering Ulster University Belfast BT15 1AP UK

**Keywords:** biofilms, extracellular exopolysaccharides, packing fraction, Payne effect, viscoelasticity

## Abstract

Biofilms are aggregated bacterial communities structured within an extracellular matrix (ECM). ECM controls biofilm architecture and confers mechanical resistance against shear forces. From a physical perspective, biofilms can be described as colloidal gels, where bacterial cells are analogous to colloidal particles distributed in the polymeric ECM. However, the influence of the ECM in altering the cellular packing fraction (ϕ) and the resulting viscoelastic behavior of biofilm remains unexplored. Using biofilms of *Pantoea sp*. (WT) and its mutant (ΔUDP), the correlation between biofilm structure and its viscoelastic response is investigated. Experiments show that the reduction of exopolysaccharide production in ΔUDP biofilms corresponds with a seven‐fold increase in ϕ, resulting in a colloidal glass‐like structure. Consequently, the rheological signatures become altered, with the WT behaving like a weak gel, whilst the ΔUDP displayed a glass‐like rheological signature. By co‐culturing the two strains, biofilm ϕ is modulated which allows us to explore the structural changes and capture a change in viscoelastic response from a weak to a strong gel, and to a colloidal glass‐like state. The results reveal the role of exopolysaccharide in mediating a structural transition in biofilms and demonstrate a correlation between biofilm structure and viscoelastic response.

## Graphical abstract

A packing fraction‐dependent transition from a weak colloidal gel to a strong colloidal gel and to a colloidal glass‐like state is reported on in a bacterial biofilm. The transition demonstrates how exopolysaccharide secretion modulates biofilm mechanical properties, a key parameter controlling their adaptability and recalcitrance in the environment.

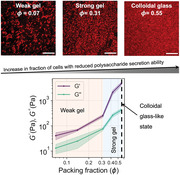

## 1 Introduction

In nature, rather than living as solitary swimmers, most bacteria live within collective communities called biofilms.^[^ ^1^, ^2^ ^]^ Upon switching to the biofilm mode of life bacteria secrete an extracellular matrix (ECM) that encapsulates the cells and acts as a protective barrier against external challenges.^[^ ^3^, ^4^ ^]^ The ECM is composed of a complex array of biological polymers, including exopolysaccharides, proteins, and extracellular DNA (eDNA).^[^ ^5^, ^6^, ^7^ ^]^ The relative abundance of these biological polymers within the ECM elicits a mechanical response between purely viscous or purely elastic material states, known as viscoelasticity.^[^ ^8^, ^9^, ^10^, ^11^ ^]^ Viscoelasticity enables biofilms to withstand large shear forces, contributing to their recalcitrance in the environment and utility as engineered living materials.^[^ ^12^, ^13^, ^14^, ^15^ ^]^ Biofilm viscoelastic response is modulated by structural components of ECM which, in turn, influence its structural properties, such as cell ordering and packing.^[^ ^11^, ^16^, ^17^ ^]^ The structural components impart physical functions to the ECM. For example, certain extracellular proteins and eDNA can cross‐link the ECM, whilst general ECM secretion creates osmotic gradients, driving colony expansion and increasing cell–cell distances.^[^ ^16^, ^18^, ^19^, ^20^ ^]^ However, a clear understanding of the relationship between biofilm viscoelastic behavior and structure as a function of ECM composition is yet to be thoroughly explored.^[^ ^21^ ^]^

Conceptually, the presence of bacterial cells (dispersed phase), ECM (continuous phase), and the interactions between the two phases within a biofilm make it structurally similar to polymer‐colloidal mixtures.^[^ ^22^, ^23^, ^24^ ^]^ This analogy has proven helpful in understanding how cell‐ECM and ECM‐ECM interactions modify the viscoelastic characteristics of plate‐grown and submerged biofilms.^[^ ^16^, ^25^ ^]^ Similarities are also observed in the case of ionic or covalent cross‐linking, which affects the elasticity and viscosity of both biofilms and polymer‐colloidal systems.^[^ ^26^, ^27^ ^]^ The physicochemical modifications that govern the structure‐rheology relationships in polymer‐colloidal systems have been thoroughly understood, enabling the engineering of abiotic materials.^[^ ^28^, ^29^ ^]^ Within such systems, factors like the particle packing fraction and polymer concentrations have been adjusted to control the viscoelasticity.^[^ ^30^, ^31^, ^32^, ^33^ ^]^ In contrast to abiotic systems, biofilms introduce a biological pathway for controlling structure and rheology through genetic manipulation.^[^ ^11^, ^13^, ^25^, ^34^ ^]^ This route has been used to study the influence of ECM composition on biofilm structure or rheology.^[^ ^16^, ^35^ ^]^ Yet, the relationship between packing fraction and biofilm viscoelasticity as a function of ECM secretion, remains largely unknown.^[^ ^14^, ^21^ ^]^

Here, we investigated the viscoelastic response of a biofilm system due to changes in its exopolysaccharide production. We used *Pantoea sp*. YR343 (WT), a biofilm‐ forming bacterium isolated from the rhizosphere of poplar.^[^ ^36^ ^]^ Some species of *Pantoea* are known to produce exopolysaccharides that promote soil aggregation and moisture retention.^[^ ^37^ ^]^ We showed that reduced production of exopolysaccharide in a *Pantoea* biofilm mutant (ΔUDP) correlated with an increase in cellular packing fraction (ϕ). Varying the ratio of WT to ΔUDP within the biofilm allowed us to selectively tune the structure of the biofilms. The structural changes coincided with a viscoelastic response that captured a transition from a weak gel to a strong gel to a glass‐like state. Using a combination of microscopy and rheometry, we revealed insights coupling the biofilm structure to its viscoelastic response during this transition. In doing so, we also revealed the existence of the Payne effect, a phenomenon shared between biofilms and model polymer colloidal systems. The presented results link biofilm structure to its rheological response.

## 2 Results and Discussion

### 2.1 Changes in Biofilm Structure Correlated with Reduction of Exopolysaccharide

**Figure 1:**
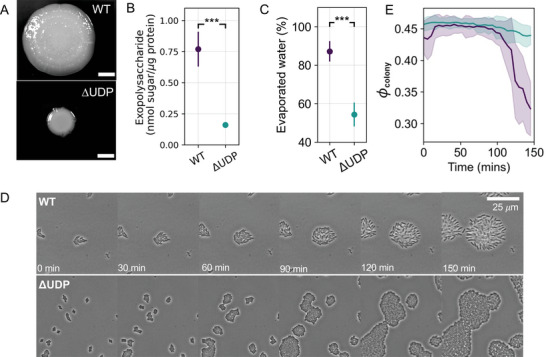
Influence of exopolysaccharide production on the structure of *Pantoea sp*. biofilms. A) Pictures of WT (top panel) and ΔUDP (bottom panel) biofilms on agar plates captured using a digital single lens reflex camera after 72 h of inoculation. B) The amount of exopolysaccharide (in *nmol* sugar/µg protein) obtained from WT and ΔUDP biofilms after 72 h of growth, using a modified phenol sulphuric method. (Sugar concentration was measured as glucose equivalents and protein concentration measured the A280 using the Nanodrop. Error bars represent one standard deviation, *n* ⩾ 3, a student's T‐test was applied to test for significance, * < 0.05, ** < 0.01, *** < 0.001). C) The evaporated water content of the WT and ΔUDP biofilms grown on 1.5% agar plates at the end of 24 h (error bars represent one standard deviation, *n* = 5, a student's T‐test was applied to test for significance, * < 0.05, ** < 0.01, *** < 0.001). D) Time‐lapse phase contrast images of WT (top panel) and ΔUDP (bottom panel) cells on an agar pad. The scale bar for all sub‐panels is 25 µm. E) A plot of biofilm's colony packing fraction ($\phi_{\mathrm{colony}}$) as a function of time for the WT and ΔUDP biofilms grown within a confined agar pad over a period of 150 minutes (error bars represent one standard deviation, *n* ⩾ 5 colonies).

Reduction of exopolysaccharide correlated with changes in colony size and evaporated water content of biofilms. The ΔUDP strain was produced via transposon mutagenesis in which the first gene (PMI39_01848) in the UDP operon encoding the biosynthetic machinery for exopolysaccharide was disrupted.^[^ ^38^ ^]^ This operon controls the synthesis and transport of an exopolysaccharide, with similarity to amylovoran, which was down‐regulated within the mutant strain.^[^ ^36^, ^38^ ^]^ To compare the macroscopic structural differences, single bacterial colonies of WT and ΔUDP were grown on agar plates for 72 h (**Figure** **1**A). Extraction experiments were performed to quantify exopolysaccharide content in the biofilms after 72 h of growth. The data show that the exopolysaccharide content in WT biofilm was five‐fold higher at 0.77 ± 0.16 *nmol* sugar/µg protein compared to the ΔUDP biofilm which had 0.14 ± 0.02 *nmol* sugar/µg protein (Figure 1B). The exopolysaccharide content was normalized to total protein content in the WT and mutant biofilm samples for comparison (see experimental section 4). From these experiments, we conclude that the production of exopolysaccharide is reduced in ΔUDP biofilms. When grown on 1.5% SOBG agar, the WT strain produced mucoid, convex colonies with small pits and a diameter of 21.5 mm (Figure 1A). In contrast, the ΔUDP mutant produced colonies that were flat, smooth, and devoid of pits with an overall diameter of 8.05 mm, three times smaller than the WT (Figure 1A). Secretion of ECM is known to influence bacterial colony sizes through the formation of osmotic pressure gradients.^[^ ^18^ ^]^ The osmotic pressure differential drives water transfer from the supporting substrate into the biofilm ECM, which is reflected in the biofilm's evaporated water content.^[^ ^18^, ^39^ ^]^ To quantify evaporated water within each biofilm, we performed drying experiments as described (experimental Section 4). The reduction of exopolysaccharide production in ΔUDP mutant resulted in a drop in the biofilm's evaporated water content to 54.3% ± 6.1% (Figure 1C), whereas the WT had an evaporated water content of 87.2% ± 5.3%. Together, these data suggested that the ΔUDP mutation resulted in smaller biofilm colonies and a lower amount of evaporated water, which correlated with a reduction of exopolysaccharide in the biofilm.

### 2.2 Exopolysaccharide Reduction Affected the Biofilm's Colony Packing Fraction

Reduction of exopolysaccharide corresponded with an increase in the colony packing fraction ($\phi_{\mathrm{colo}\mathrm{ny}}$) of ΔUDP biofilms. To understand the impact of exopolysaccharide reduction on the structural evolution of the biofilms, we performed time‐lapse microscopy on 2D colonies grown on agarose pads (Figure 1D). From these experiments, we calculated the biofilm's $\phi_{\mathrm{colony}}$ (Figure 1E), which was defined as the area fraction of cells within a colony perimeter (Experimental Section 4; Figure S1, Supporting Information). At initial times (*t* < 100 mins), both the WT and ΔUDP biofilms had a comparable microstructure with 0.43 ⩽$\phi_{\mathrm{colony}}$⩽ 0.48. Both strains displayed colony microstructure resembling a jammed state. A jammed state is one where movement is constricted by neighboring cells and it occurs at high packing fraction values.^[^ ^40^ ^]^ As the WT biofilm colonies increased in size (*t* > 100 mins), an increase in the cell–cell distance was observed, which reduced the average colony packing fraction to $\phi_{\mathrm{colony}}$ = 0.325 (Figure 1D, E; Movie S1, Supporting Information). In contrast, the cell clusters from ΔUDP colonies remained in a jammed state $\phi_{\mathrm{colony}}$ ≈ 0.44 for the duration of the experiment (Figure 1D, Movie S2, Supporting Information). At later time points (*t* ≈ 150 min) the differences in biofilm's $\phi_{\mathrm{colo}\mathrm{ny}}$ between WT and ΔUDP biofilm was reminiscent of viscoelastic liquid/gel to glass‐like transition observed in colloidal systems.^[^ ^41^, ^42^ ^]^ Such structural changes alter the viscoelastic response of colloidal systems. We therefore wanted to investigate how biofilm viscoelasticity is modified due to the observed transition in biofilm structure.

### 2.3 Exopolysaccharide Reduction Correlated with a Rheological Transition

**Figure 2:**
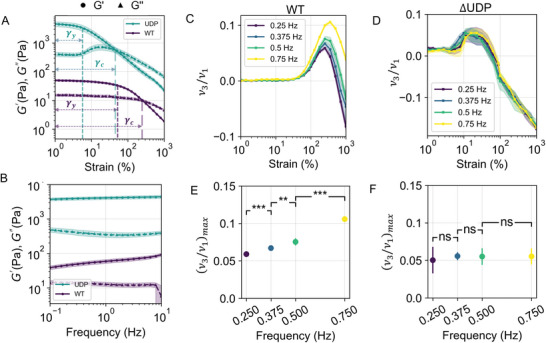
Reduction of exopolysaccharide production affected the rheology of WT and ΔUDP biofilms. A) Variation in elastic modulus ($G^{{}^{\prime }}$, filled circle) and viscous modulus ($G^{{}^{\prime \prime }}$, filled triangle) as a function of applied γ at 0.5 Hz. $\gamma_{y}$ denotes the value of yield strain below which the viscoelastic response is considered to be linear. Above $\gamma_{y}$ nonlinear effects appear in the rheological response. (described in Figure S2, Supporting Information). $\gamma_{c}$ is the cross‐over strain beyond which viscous response dominates the elastic response in the biofilms. B) Variation in $G^{{}^{\prime }}$ and $G^{{}^{\prime \prime }}$ as a function of applied frequency at γ=1% . C) Plot of the intracyle thickening (ν_3_/ν_1_) (ratio of third to first order viscous Chebyshev coefficients) for WT as a function of applied strain. D) Plot of the intracyle thickening (ν_3_/ν_1_) for ΔUDP as a function of applied γ. E) Statistical significance of the (ν_3_/ν_1_)_*max*_ (maximum thickening ratio at a given frequency) for the WT biofilms (*n* ⩾ 3). A paired student t‐test was applied, (* < 0.05, ** < 0.01, *** < 0.001) F) Statistical significance of the (ν_3_/ν_1_)_*max*_ (maximum thickening ratio at a given frequency) for the ΔUDP biofilms (*n* ⩾ 3). A paired student t‐test was applied, (* < 0.05, ** < 0.01, *** < 0.001).

Reduction in exopolysaccharide production correlated with an increase in the biofilm elasticity by two orders of magnitude. To probe the viscoelastic behavior of the WT and ΔUDP strains, we performed oscillatory shear rheometry tests. Both biofilm strains displayed an elastically dominated response where the elastic modulus ($G^{{}^{\prime }}$) was greater than the viscous modulus ($G^{{}^{\prime \prime }}$), in the linear viscoelastic regime (strain(γ)<7%). The WT strain had a plateau $G^{\prime }$ of 58 ± 5 Pa, while plateau $G^{\prime }$ for the ΔUDP biofilm measured 4450 ± 550 Pa, approximately 2 orders of magnitude higher. The plateau $G^{{}^{\prime \prime }}$ of the WT strain was 12.7 ± 1.5 Pa whereas the ΔUDP biofilm measured 360 ± 55 Pa, aprroximately 1.25 orders of magnitude higher than the WT (Figure 2A). The plateau phase angle for the WT strain was 17.15° ± 1.22°, whilst for the ΔUDP mutant it was 5.06° ± 0.11° (Figure S2, Supporting Information). These data indicated that the WT strain had a proportionally larger viscous component contributing to its viscoelastic response compared to the ΔUDP strain.

A reduction in exopolysaccharide correlated with a distinct transition in viscoelastic behavior. The onset of nonlinearity in the rheological behavior of biofilms is quantified by the yield strain ($\gamma_{y}$). Beyond $\gamma_{y}$ the biofilm structure rearranges and cannot return to its original state. For the WT, $\gamma_{y}$ was 55%, whilst $\gamma_{y}$ for the ΔUDP mutant was 6% (**Figure** **2**A). Beyond $\gamma_{y}$, both biofilms exhibited different trends in $G^{{}^{\prime \prime }}\left(\gamma \right)$ with increasing γ. The shape of $G^{{}^{\prime \prime }}\left(\gamma \right)$ indicated the nature of viscous dissipation occurring within the biofilm due to structural rearrangement (Figure 2A). The ΔUDP biofilm displayed a weak strain overshoot indicated by the local increase in $G^{{}^{\prime \prime }}\left(\gamma \right)$, which began at γ = 6 % and peaked at γ = 20 %. The peak viscous modulus was found to be equal to $G^{{}^{\prime \prime }}$ = 708 ± 107 Pa, approximately two times larger than the plateau $G^{{}^{\prime \prime }}$ (Figure 2A). Weak strain overshoots have been frequently reported in colloidal glasses/gels^[^ ^43^ ^]^ and have also been observed in biofilms of *Pseudomonas aeruginosa*, *Bacillus subtilis*, and *Vibrio cholerae*.^[^ ^11^, ^16^ ^]^ In contrast, the WT strain was devoid of weak strain overshoots and had $G^{{}^{\prime \prime }}\left(\gamma \right)$ behavior indicative of strain thinning that has been commonly seen in viscoelastic liquids and polymer melts.^[^ ^43^ ^]^

Finally, a reduction in the exopolysaccharide eliminated frequency‐dependent thickening in biofilms. We measured the frequency‐dependent behavior of the WT and ΔUDP biofilms using frequency sweeps (ω = 0.1 − 10 Hz). Curves of $G^{{}^{\prime }}\left(\omega \right)$ and $G^{{}^{\prime \prime }}\left(\omega \right)$ were non intersecting across the frequency range (Figure 2B) for both biofilms. The WT biofilm's $G^{{}^{\prime }}\left(\omega \right)$ exhibited a weak frequency dependence (slope ≈ 0.187), whilst the ΔUDP biofilm showed frequency independence (slope ≈ 0.0365).^[^ ^44^ ^]^ Observation of such scaling in log–log plots suggests a commonality of biofilms with colloid‐polymer mixtures or colloidal gels, which also display a $G^{{}^{\prime }}\left(\omega \right)$ frequency dependence.^[^ ^30^, ^45^ ^]^ We then measured the frequency dependence of each biofilm at large strain amplitudes. The Chebyshev framework implemented in MITLaos software was used to analyze the nonlinear thickening/thinning behavior by quantifying the intracycle thickening ratio ν_3_/ν_1_ (defined as the ratio of third to first order Chebyshev viscous coefficients) (Figure 2C,D). The intracycle thickening ratio for the WT strain demonstrated frequency dependence (Figure 2E). In contrast, the ΔUDP biofilm displayed frequency independence of thickening/thinning ratio across the frequency range (Figure 2F). This result indicated the contribution of exopolysaccharide in altering the nonlinear thickening characteristic of biofilms. The presence of exopolysaccharide enabled the WT biofilm to relax back to its original state. In contrast, ΔUDP biofilm exhibited a frequency invariant intracycle thickening response and dissipated a larger amount of energy compared to WT (Figure S3, Supporting Information) due to a reduction of exopolysaccharide in the biofilm. Taken together these rheological experiments lead us to conclude that the WT strain displayed behavior of a colloidal gel, whilst the ΔUDP mutant displayed glass‐like behavior. WT and ΔUDP biofilms represented the two limiting cases for low and high ϕ, therefore we wanted to investigate how biofilm rheology and structure transitions at intermediate packing fractions.

### 2.4 Evaporated Water Content and Packing Fraction Were Controlled Using a Co‐Culturing Approach

**Figure 3:**
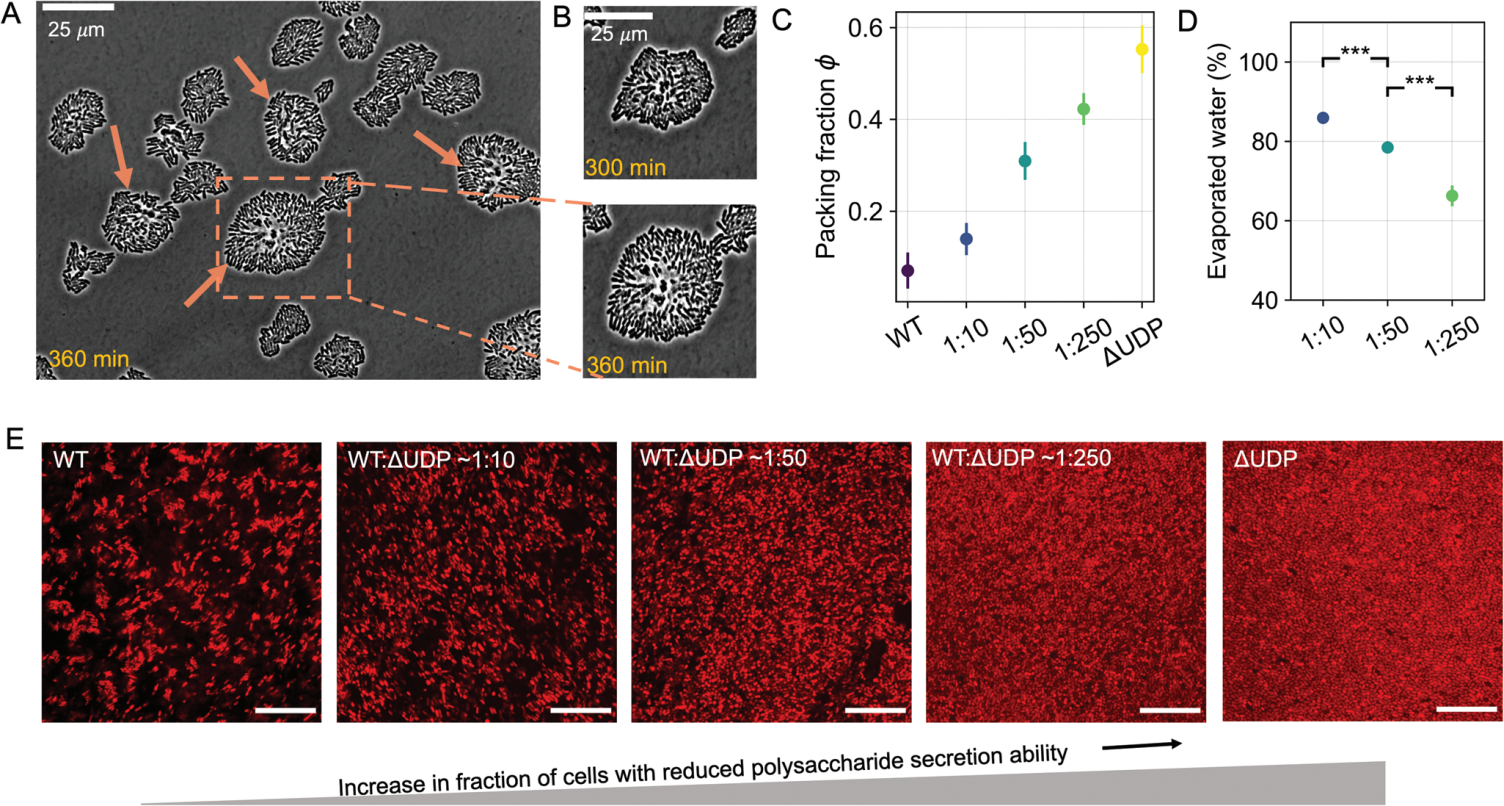
Ratio of WT to ΔUDP altered the structure and evaporated water content of co‐cultured biofilms. A) Representative biofilms of WT and ΔUDP co‐cultures after 6 h on an agar pad. Red arrows indicate the location of the colony in which WT cells are surrounded by densely packed ΔUDP cells. B) (top) Zoom‐in view of a mixed *Pantoea sp*. colony and (bottom) shows the same field of view 60 min later. ECM secreted from the center of cellular cluster reduced the local packing density of colonies. C) Calculated ϕ of the *Pantoea sp*. biofilms with different inoculation ratios of WT to ΔUDP grown on an agar plate for 48 h. D) Evaporated water content of the co‐cultured biofilms after 24 h of growth (error bars represent one standard deviation, n=5, a student's T‐test was applied to test for significance, * < 0.05, ** < 0.01, *** < 0.001). E) Representative confocal images of biofilms 10 µ*m* from the coverslip surface after 48 h of growth, which were started from different inoculation ratios of WT to ΔUDP. Decreasing the ratio of WT caused reduction of exopolysaccharide, transitioning the structure of biofilm into a jammed state.

Modulating the co‐culture ratios of the WT and ΔUDP strains allowed control over biofilm structure. To visualize the changes in structure of the co‐cultured biofilms, we performed time‐lapse imaging in 2D on confined agarose pads with a 1:10 ratio of WT to ΔUDP (**Figure** **3**A). Initially, the co‐cultured biofilm displayed a jammed structure similar to the mono‐cultured biofilms of ΔUDP (Figure 3B‐top panel). As the WT began to produce exopolysaccharide, the packing at the center of the co‐cultured colony reduced (Figure 3B‐bottom panel). At the end of the experiment (*t* = 6 h) the biofilm structure consisted of exopolysaccharide rich 'islands' which were surrounded by clusters of jammed ΔUDP cells (Figure 3B‐ bottom panel, Movie S3, Supporting Information). The agar pad setup led us to conclude that exopolysaccharide production is an important factor in structuring the colonies of co‐cultured biofilms. To establish the relationship between ϕ and viscoelastic response, we co‐cultured WT and ΔUDP biofilms at different inoculation ratios on agar plates. Agar plates enabled the spreading of the biofilm in 3D, removing the constraint in the vertical dimension imposed by the 2D agarose pad setup. These biofilms were then collected and transferred for optical and rheological characterization, enabling us to directly correlate ϕ with the biofilm viscoelastic response.

Increasing the co‐culture ratio of the ΔUDP mutant increased the ϕ (Figure 3C) and decreased the biofilm's evaporated water content (Figure 3D). The co‐cultured biofilms were grown for 48 h, then transferred and imaged using a confocal microscope (Figure 3E). The confocal slices were used to compute ϕ, defined as the ratio of the area occupied by cells to the total image area (Figure S4, Supporting Information). By increasing the ΔUDP ratio in co‐cultured biofilms exopolysaccharide production within the biofilms was reduced. This was reflected in the packing fraction which increased from ϕ = 0.07 ± 0.04 to ϕ = 0.42 ± 0.03 (Figure 3C). At the highest co‐culture ratio (WT to ΔUDP ≈1: 250) the biofilm had a reduced amount of exopolysaccharide in the matrix and approached a jammed structure. The mono‐cultured ΔUDP biofilms had a packing fraction of ϕ = 0.55 ± 0.05 which approached the theoretical random packing limit for rods/ellipsoids $\phi_{\mathrm{rcp}}$ ≈ 0.64.^[^ ^46^, ^47^ ^]^ We therefore concluded that tuning the co‐culture ratio allowed control over the amount of exopolysaccharide and influenced the ϕ of co‐cultured biofilms. Using this approach, we further investigated how the reduction of exopolysaccharide affected the ϕ and consequently the biofilm rheological behavior.

### 2.5 Reduction of Exopolysaccharide Altered the Packing Fraction and Rheology of Biofilms

**Figure 4:**
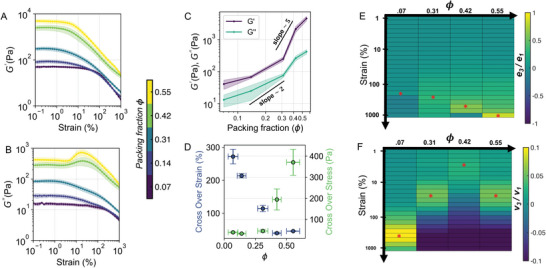
Reduction of exopolysaccharide correlated with changes in the viscoelastic response of biofilms. A) Variation of $G^{\prime }$as a function of the γ on a log–log plot. Color codes represent the ϕ of biofilms (error bars represent the standard deviation of the mean, n⩾3) B) $G^{\prime \prime }$as a function of γ on a log–log plot (error bars represent standard deviation of the mean, n⩾3) C) Plot of linear $G^{\prime }$ and linear $G^{\prime \prime }$as a function of ϕ. Lines with a slope of *two* and *five* are shown for reference. (error bars represent the standard deviation of the mean, n⩾3) D) Plot of cross‐over stress (σ_*c*_) and cross‐over strain ($\gamma_{c}$) as a function of the biofilm's ϕ. (error bars represent the standard deviation of the mean, n⩾3) E) A heat‐map of mean stiffening ratio (*e*_3_/*e*_1_, ratio of third to first order elastic Chebyshev coefficients) as a function of γ and ϕ at 0.75 Hz. Stars denote the location of the maximum value of *e*_3_/*e*_1_ at a constant ϕ (n⩾3, standard deviation heat maps are included in Figure S5A, Supporting Information). F) A heat‐map of mean thickening ratio (ν_3_/ν_1_) as a function of γ and ϕ at 0.75 Hz. Stars denote the location of the maximum value of ν_3_/ν_1_ at a constant ϕ (n⩾3, standard deviation heat maps are included in Figure S5B, Supporting Information).

Elastic modulus exhibited a two‐step scaling relationship as a function of packing fraction. To quantify the relationship between changes in ϕ and biofilm viscoelasticity we performed amplitude sweeps (**Figure** **4**A, B). An increase in the ϕ in co‐cultured biofilms resulted in an increase in the magnitude of the linear $G^{{}^{\prime }}$ (Figure 4A). Cumulatively, a seven‐fold increase in ϕ resulted in two orders of magnitude increase in the linear $G^{\prime }$, whilst the linear $G^{\prime \prime }$ increased by an order of magnitude (Figure 4C). Within the packing fraction range (0.07 ⩽ ϕ ⩽ 0.31) the linear $G^{\prime }$ scaled as ($G^{{}^{\prime }}\approx \;\phi^{\mu }$) with a slope of µ = 1.24. Due to the relatively small increase in elasticity, biofilms within this range of packing fraction values (0.07 ⩽ ϕ ⩽ 0.31) can be termed as weak gels. Above the packing fraction value (ϕ > 0.31) a slope of µ = 5.11 is observed, indicating a rapid rise in elasticity, a regime characteristic of strong gels. The two‐step scaling signified a transition from a weak to a strong gel state; brought about by reduction of exopolysaccharide within the biofilms .

Reduction of exopolysaccharide altered the viscous dissipation behavior in biofilms. Changes in $G^{{}^{\prime \prime }}$ due to γ provided insights into the energy dissipation mechanisms, which occur as the biofilm structure is deformed. A log‐log plot of $G^{{}^{\prime \prime }}\left(\gamma \right)$ for the biofilms with 0.14 ⩽ ϕ ⩽ 0.31 exhibited two power law gradients. For strain amplitudes between 8%≤γ≤100% a gradient of −0.284 was measured, whilst between 100%≤γ≤1000% a gradient of −0.559 was measured (Figure S6A, Supporting Information). The emergence of two power‐law decays indicated that two different energy dissipation mechanisms were present within the co‐cultured biofilms. For biofilms with ϕ > 0.4, a weak strain overshoot $G^{{}^{\prime \prime }}\left(\gamma \right)$ in amplitude sweep was observed (Figure 4B). The magnitude of the strain overshoot ratio ($G_{peak}^{{}^{\prime \prime }}$/$G_{plateau}^{{}^{\prime \prime }}$) was smaller in the co‐cultured biofilm $G_{peak}^{{}^{\prime \prime }}$/$G_{plateau}^{{}^{\prime \prime }}$ = 1.36 when compared to the mono‐cultured ΔUDP biofilm $G_{peak}^{{}^{\prime \prime }}$/$G_{plateau}^{{}^{\prime \prime }}$ = 1.76 (Figure S6B and S6C, Supporting Information). The $\gamma_{c}$ defined as intersection of $G^{{}^{\prime }}$ and $G^{{}^{\prime \prime }}$, for the co‐cultured biofilms decreased with increasing ϕ (Figure 4D). This behavior and the weak strain overshoot are characteristic of the Payne effect.^[^ ^48^, ^49^, ^50^ ^]^ The $G^{\prime \prime }$ behavior in these systems is linked to the packing fraction of filler particles within the polymeric network. At intermediate strain amplitudes the network began to break down into clusters giving rise to inter‐cluster hydrodynamic interactions. The interaction manifested as a local increase in $G^{\prime \prime }$ at intermediate strain (γ = 20%) for our biofilm system, which is in line with published observations.^[^ ^51^, ^52^ ^]^ Together the co‐cultured biofilms for which ϕ > 0.4 and the mono cultured ΔUDP for which ϕ = 0.55, exhibited rheological behavior characteristic of materials that display the Payne Effect.^[^ ^50^ ^]^ The different characteristic shapes of $G^{{}^{\prime \prime }}$ as a function of γ seen at low (ϕ < 0.14), intermediate (0.14 < ϕ < 0.42) and high (0.42 ⩽ ϕ ⩽ 0.55) packing fractions demonstrated how the viscoelasticity of our biofilm system altered with ϕ.

Large amplitude shear experiments captured changes in intracycle thickening and thinning in biofilms related to the reduction of exopolysaccharide. To characterize the behavior of mono and co‐cultured biofilms at large strains, we utilized nonlinear viscoelastic measures developed using the framework of Chebyshev polynomial analysis. The maximum value of intracycle elastic Chebyshev coefficient ((*e*_3_/*e*_1_)_*max*_) increased with ϕ and the maximum occurred at progressively higher strain amplitudes (indicated by stars in Figure 4E). We posit that a reduction of the exopolysaccharide content resulted in increased strain stiffening of the ECM‐cell network with applied strain. The heat‐map for intracycle viscous Chebyshev coefficient (ν_3_/ν_1_) showed maximum intracycle thickening (ν_3_/ν_1_)_*max*_ at γ=354% for the WT biofilms (ϕ = 0.07). For the ΔUDP biofilms (ν_3_/ν_1_)_*max*_ had a value of 0.056 ± 0.0043 at strain amplitude of γ=31.5%. At intermediate packing fractions (0.1 < ϕ < 0.5), the value of maximum intracyle thickening ratio showed a decreasing trend (Figure S7, Supporting Information) and the strain where maximal intracycle thickening occurred is also lowered (indicated by stars in Figure 4F). We attributed this behavior to the reduction in exopolysaccharide and evaporated water content within the biofilm, which reduced the viscous dissipation within the ECM. Hence the observed decreasing trend of maximum intracycle thickening (ν_3_/ν_1_)_*max*_ as a function of packing fraction (Figure 4F; Figure S7, Supporting Information). For ϕ ⩾ 0.42, the increase in maximum intracycle thickening can be attributed to cellular crowding (also known as caging) and an increased probability of short‐ranged cell–cell attractive interactions. Overall, the large strain experiments suggested that the exopolysaccharide in co‐cultured biofilm matrix contributed toward intracycle stiffening and thickening behavior. Therefore, tuning the co‐culture ratios can be used to control the ϕ and the resulting linear and nonlinear rheological characteristics of the biofilm system.

## 3 Conclusion

Our results showed that a genetic mutation to the UDP operon in *Pantoea sp*. resulted in a five‐fold reduction in exopolysaccharide production in the ΔUDP biofilm when compared to the WT biofilm. The reduction of exopolysaccharide correlated with an increase in the biofilm's ϕ leading to a glass‐like state. Using time‐lapse and confocal microscopy, we quantified the changes in ϕ and performed drying experiments to measure the evaporated water content of the WT biofilm and its exopolysaccharide reduced mutant. Co‐culturing of the strains allowed us to tune the exopolysaccharide content within the biofilm, allowing control over their ϕ. The approach enabled us to map the structure‐rheology relationships and relate the biofilm's packing fraction to its viscoelastic response, revealing a two‐step elastic and viscous modulus dependence upon ϕ. Using advanced rheometry techniques, we probed a range of viscoelastic measures, revealing the existence of the Payne effect and the role of exopolysaccharide reduction in mediating the intracycle stiffening or thickening behavior in our biofilm system.

The presented results demonstrated that exopolysaccharide production influenced the macro‐scale colony diameter, micro‐scale structure and rheology in bacterial biofilms. While exopolysaccharides have been recognized for their role in surface adhesion and altering cellular ordering in biofilms,^[^ ^16^, ^53^, ^54^ ^]^ their role in changing ϕ and rheology has received limited attention.^[^ ^17^ ^]^ Consequently, the relationship between ϕ, exopolysaccharide concentration and biofilm viscoelasticity has remained elusive. We addressed this shortcoming by using a co‐culturing approach, which allowed us to regulate the exopolysaccharide concentration in the biofilms. This allowed us to control the biofilm ϕ and reveal a transition from a weak to a strong gel state. In doing so, our experiments have confirmed the presence of packing fraction‐dependent increases in elasticity, which is a well‐known feature of polymer‐colloidal systems.^[^ ^30^, ^31^, ^32^, ^33^ ^]^ The origin of this relationship could be attributed to the increasing spatial density of bacteria in comparison to the ECM, which have elastic moduli on the order of 0.1 − 1 MPa and 40 Pa, respectively.^[^ ^55^, ^56^ ^]^ We anticipate that this behavior will be applicable across biofilms of different species. However, the composition and properties of the matrix biopolymers will influence the scaling relationship between $G^{\prime }$ and ϕ. As has been observed with polymer‐colloidal systems, we speculate that exopolysaccharide concentration and biopolymer crosslinking density play a role in this scaling.^[^ ^57^ ^]^

The relationship between ϕ and viscoelastic response as explored in this manuscript suggested that biofilms can exhibit variations in viscoelastic behavior as a function of cell density. Our results suggest a link between exopolysaccharide secretion and the viscoelastic response of biofilms in large shear environments. Biofilm colonies developing in shear flow exhibit gradients of ϕ and the magnitude of shear forces can induce a transition from hemispherical shape to elongated colony shapes.^[^ ^25^, ^58^ ^]^ Prior studies, particularly those concentrating on the nucleation of biofilm streamers and colony deformation have also hinted at heterogeneity in ϕ.^[^ ^25^, ^59^ ^]^ Furthermore, shear stress‐dependent increases in cell density have previously been documented in biofilms growing on surfaces of annular reactors.^[^ ^60^, ^61^ ^]^ Therefore, understanding packing fraction‐dependent rheology could assist in predicting the shear stress profiles that induce streamer nucleation, drive biofilm colony morphogenesis, and control bioreactor stability. An emerging application of biofilm viscoelasticity lies in the realm of 3D printing, particularly in developing engineered living materials.^[^ ^62^ ^]^ Formulating relationships between ϕ and rheology can offer scientists increased flexibility in manipulating the nematic ordering of cells, patterning cells at varying densities, and printing structures with graded stiffness, resulting in the development of novel functional bioinspired materials.^[^ ^15^, ^63^ ^]^

While we expect our findings to be generally applicable across biofilms, it is important to be aware that the expression of exopolysaccharide in biofilms is substrate and species‐dependent.^[^ ^64^ ^]^ Consequently, the range of ϕ that can be explored may be influenced by the specific experimental systems utilized. Furthermore, unlike model colloidal systems where polymer and particle concentrations can be controlled independently,^[^ ^45^ ^]^ our experimental system only allowed us to reduce the amount of exopolysaccharide in biofilm and quantify the emergent structure‐rheology relationships. Although the presented work has focused on the polysaccharide component of ECM, it is important to note that cell‐cell and cell‐matrix interactions mediated by proteins and eDNA also play significant roles. For instance, recent reports have suggested that cell–cell protein interactions can extend the strain‐stiffening region.^[^ ^21^ ^]^ Consequently, we anticipate variations in the scaling of biofilm ϕ and viscoelastic modulus for biofilms that are either protein‐dominant or cross‐linked by eDNA. Uncovering these scaling relationships could inform the design and use of biofilms as rheologically tailored living materials.

## 4 Experimental Section

### Bacterial Strains and Mutants

*Pantoea sp*. YR343 , a root colonizing, gram‐negative, motile bacterium was isolated from the rhizosphere of *Populus deltoides*. Transposon mutagenesis was performed on *Pantoea sp*. YR343 using plasmid pRL27, which encoded a mini‐Tn5 transposon. The process generated a library of mutants including ΔUDP (also known as UDP::Tn5), which exhibited altered exopolysaccharide synthesis capabilities.^[^ ^36^, ^38^ ^]^ Glycerol stocks of WT and ΔUDP strains were prepared and stored in −80 °C.

### Bacteria and/or Biofilm Growth

Sterile SOBG medium containing 20 g tryptone, 5 g yeast extract, 0.5 g NaCl, 2.4 g MgSO_4_, 0.186 g KCl, 50 ml of 40% v/v glycerol in 1 L of milli‐Q water was prepared. SOBG agar was obtained by adding 15 g of agar to the SOBG medium followed by an autoclave cycle. Bacteria from frozen glycerol stocks at −80 °C were streaked out on SOBG agar. Single colonies of WT and ΔUDP strains were inoculated in sterile SOBG medium and grown overnight in a shaking incubator (150rpm) at 24 °C. Appropriate volumes of overnight cultures were pipetted into conical tubes to obtain 1: 0, 1: 10, 1: 50, 1: 250, 0: 1 ratios of WT to ΔUDP and were subsequently mixed by vortexing. 150 µL of these cultures were then transferred on the sterile agar plates and spread using an L‐shaped spreader. The agar plates were incubated at 24 °C for 48 h, which allowed biofilms to develop. The developed biofilms were used for confocal microscopy or rheometry experiments.

### Microscopy and Image Analysis

Time‐lapse images were acquired on a Nikon Ti‐s microscope operating in phase contrast mode with a 60X oil (NA = 1.4, Plan Apochromat) immersion objective. 1.5% agarose pads made from M9 minimal media, supplemented with 0.5% glucose, were prepared within 25 µ*L* gene frames attached to #1.5, 25 mm x 60 mm coverslip. Overnight bacterial cultures grown in SOBG medium were diluted to 0.1 optical density in M9 media. Subsequently, 1 µ*L* of diluted culture was transferred onto the agarose pad and sealed with a 22mm X 22 mm coverslip (confined geometry). Images were acquired at 5 min intervals for 6 h at a magnification of 60X. Acquired images were segmented using the Python package cellpose. Biofilm's colony packing fraction ($\phi_{\mathrm{colo}\mathrm{ny}}$) was calculated as the area fraction of cells within a colony perimeter. The colony area was calculated using the region‐props function in Python (Figure S1, Supporting Information).

Confocal imaging was performed on biofilms grown on agar plates (unconfined geometry). Biofilms were stained with 5 µL of Syto 63 (nucleic acid stain) and FM 4‐64 FX (cell membrane stain), diluted to 1: 1000 in Tris buffer. Stained biofilms were collected and placed within 25 µ*L* gene frames and sealed with a #1.5 coverslip on top. Confocal images were acquired using a Leica SP8 inverted confocal with a 100X oil immersion lens (NA = 1.4). A pinhole size of one airy unit and an excitation wavelength of 660 nm was used. Images were processed using ImageJ and an in‐house Matlab script to obtain ϕ, which was defined as the ratio of the area occupied by cells to the total image area (Figure S4, Supporting Information).

### Evaporated Water Measurement

Overnight cultures of WT and ΔUDP, respectively, were mixed in ratio of 1:0, 1:10, 1:50, 1:250, 0:1. 50 µl of the mixed cultures were pipetted on 1.5% SOBG agar and incubated at 24 °C for 24 h for biofilms to develop. For each ratio of mixed cultures, *n* = 5 biofilm replicates were grown. Mass of empty weighing boats (*m*_*e*_) was recorded and biofilm samples were scraped onto each boat. This enabled quantification of the total wet mass (*m*_*t*_) of the biofilm. The weighing boats were incubated at 70 °C for 3 h. After water had evaporated the mass of system (*m*_*d*_) was recorded again. Evaporated water content (*EW*) was calculated based on the formulae *EW* =$\frac{\left(m_{t}-m_{e}\right)-\left(m_{d}-m_{e}\right)}{\left(m_{t}-m_{e}\right)}$.

### Rheometry

Biofilm rheology was measured using a Kinexus Pro+ rheometer operating in strain control mode with a 20 mm diameter plate‐plate geometry. Slip was minimized by attaching adhesive‐backed grit paper (#120) to the top and bottom plates. Biofilm samples were collected from the agar plates and pooled on the bottom rheometer plate. This approach had been shown to cause differences in the magnitude of elastic and viscous modulus, however, the difference was much lower in agar‐grown biofilms compared to submerged biofilms.^[^ ^8^, ^65^ ^]^ In both approaches, the evolution of elastic and viscous behavior as a function of strain remained unchanged. The top plate was lowered to a gap height of 1 mm and the normal force was set to 0.1 N. A solvent trap was used to prevent sample dehydration during measurement. Frequency sweeps were performed at a strain amplitude of 1% for WT and ΔUDP biofilms. Amplitude sweeps were performed for strain amplitudes ranging from 1%‐1000% while holding the frequency constant at 0.5 Hz for WT to ΔUDP biofilms in the ratio of 1: 0, 1: 10, 1: 50, 1: 250, 0: 1. Large Amplitude Oscillatory Shear (LAOS) experiments were performed for strain amplitudes ranging from 1%‐1000% while holding the frequency constant at 0.25, 0.375, 0.5, or 0.75 Hz for WT to ΔUDP biofilms in the ratio of 1: 0, 1: 50, 1: 250, 0: 1. A decimator setting of  5 for the ΔUDP and 30 for the WT allowed us to capture distortion‐free strain waveforms during LAOS experiments. The recorded LAOS waveforms were checked for stability, truncated to five cycles, and then input into MITLaos^[^ ^66^ ^]^ for obtaining the intracycle stiffening (*e*_3_/*e*_1_) and intracycle thickening (ν_3_/ν_1_) ratios. A positive value of *e*_3_/*e*_1_ indicated stiffening within the material, while a negative value indicated softening. Similarly, positive or negative values of ν_3_/ν_1_ indicated thickening or thinning in the material.

### Exopolysaccharide Quantitation

Extraction and quantitation of exopolysaccharides was performed as described previously with some modifications.^[^ ^67^, ^68^ ^]^ Briefly, the WT and ΔUDP mutant of *Pantoea sp*. YR343 were streaked onto SOBG agar plates and grown at 25 °C for 72 h. To each plate, enough PBS was added to facilitate scraping cells off of the plate and collection into a sterile 50 mL tube. The final volume for each sample was 12.5 mL of resuspended cells. Then each sample was vortexed at high speed for 1 min, incubated with shaking at 30 °C for 1.5 h. The vortex and incubation step was repeated once, then a small aliquot was taken for total protein measurements to normalize the cell numbers between samples. Next, the samples were centrifuged at 10,000 x *g* for 15 min to remove the cells and the supernatant containing the ECM components was placed into a fresh tube with 200 µgml^‐1^ proteinase K and incubated overnight at 37 °C with shaking. The next day, the supernatant samples were extracted with phenol‐chloroform, and the top layer was removed to a fresh tube. To this tube a volume equal to three times the sample volume of 95% ethanol was added before samples were placed at −20 °C for at least overnight to allow for exopolysaccharide precipitation. Precipitated exopolysaccharides were collected by centrifugation at 10,000 × *g* for 15 min and the supernatant was removed. Pellets were then resuspended in two milliliters of nanopure water. Measurement of exopolysaccharides was performed using the phenol‐sulfuric acid method adapted for the microplate reader with a standard curve determined for glucose in order to quantify nmol of sugars.^[^ ^69^ ^]^ Measurements of total protein were performed by lysing pellets from 1 mL samples collected before exopolysaccharide extraction using B‐PER II Protein Extraction Reagent (Thermo Scientific) and measuring the A280 using the Nanodrop. Exopolysaccharide content is shown as *nmol* sugars/µg protein to normalize for cell number in WT and mutant biofilm samples.

### Growth Curve Measurement

Cultures of WT and ΔUDP were prepared in Luria‐Bertani media and grown overnight. The next day, a 24‐well plate was filled with 1 mL SOBG media per well. Wells were inoculated by adding 10 µL from an overnight culture to the appropriate well. Each sample had five replicates with four wells serving as blank controls. Once inoculated, the plate was placed into an Agilent (formerly BioTek) Cytation 5 plate reader and the absorbance at 600 nm was measured every hour for 24 h (Figure S8, Supporting Information).

### Statistical Analysis

Data presented in this manuscript were processed using Python, Matlab and Microsoft Excel. Results were presented as the mean and standard deviation of the mean. n⩾3 samples were used to calculate the statistical measures. A student's T‐test was applied to test for significance, * < 0.05, ** < 0.01, *** < 0.001.

## Conflict of Interest

The authors declare no conflict of interest.

## Supporting information

Supporting Information

Supplemental Movie 1

Supplemental Movie 2

Supplemental Movie 3

## Data Availability

The data that support the findings of this study are available from the corresponding author upon reasonable request.
