# Supplementary material for: Microstructural and Rheological Transitions in Bacterial Biofilms
Source: Adv Sci (Weinh). 2023 Jul 31;10(27):2207373. doi: 10.1002/advs.202207373 (PMC10520682; doi:10.1002/advs.202207373)
Supplement: Supplementary file 1 — Supporting Information [file ADVS-10-2207373-s001.pdf]

ADVANCED SCIENCE
Open Access

Supporting Information

for *Adv. Sci.*, DOI 10.1002/advs.202207373

Microstructural and Rheological Transitions in Bacterial Biofilms

*Samuel G.V. Charlton, Amber N. Bible, Eleonora Secchi, Jennifer L. Morrell-Falvey, Scott T. Retterer, Thomas P. Curtis, Jinju Chen and Saikat Jana**

Supporting information: Microstructural and rheological transitions in bacterial biofilms

*Samuel G.V. Charlton, Amber N. Bible, Eleonora Secchi, Jennifer L. Morrell-Falvey, Scott T. Retterer, Thomas P. Curtis, Jinju Chen, Saikat Jana**

Dr. Samuel G.V. Charlton
Address
Department of Civil, Environmental and Geomatic Engineering, Institute of Environmental Engineering, ETH Zurich, Zurich, Switzerland
Address
School of Engineering, Newcastle University, Newcastle Upon Tyne, UK
Email Address: charlton@ifu.baug.ethz.ch

Dr. Amber N. Bible
Address
Biosciences Division, Oak Ridge National Laboratory, Oak Ridge, TN, USA
Email Address: biblean@ornl.gov

Dr. Eleonora Secchi
Address
Department of Civil, Environmental and Geomatic Engineering, Institute of Environmental Engineering, ETH Zurich, Zurich, Switzerland
Email Address: esecchi@ethz.ch

Dr. Jennifer L. Morrell-Falvey
Address
Biosciences Division, Oak Ridge National Laboratory, Oak Ridge, TN, USA
Email Address: morrelljl1@ornl.gov

Dr. Scott T. Retterer
Address
Biosciences Division, Oak Ridge National Laboratory, Oak Ridge, TN, USA
Address
Center for Nanophase Material Sciences, Oak Ridge National Laboratory, Oak Ridge, TN, USA
Email Address: rettererst@ornl.gov

Prof. Thomas P. Curtis
Address
School of Engineering, Newcastle University, Newcastle Upon Tyne, UK
Email Address: tom.curtis@newcastle.ac.uk

Dr. Jinju Chen
Address
School of Engineering, Newcastle University, Newcastle Upon Tyne, UK
Email Address: jinju.chen@newcastle.ac.uk

Dr. Saikat Jana
Address
School of Engineering, Ulster University, Belfast, UK
Address
School of Engineering, Newcastle University, Newcastle Upon Tyne, UK
Email Address: s.jana@ulster.ac.uk

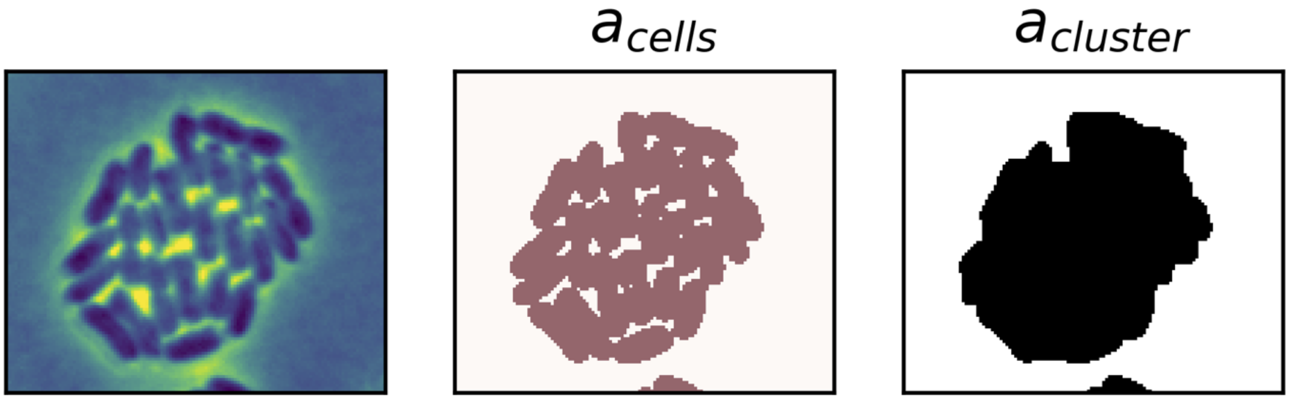

$$\phi_{colony} = \frac{a_{cells}}{a_{colony}}$$

Figure S1: Procedure for calculating colony packing fraction of biofilms grown on confined agar pads. (A) Shows the phase contrast image of a colony on an agar pad. (B) Shows the cells that have been recognised based on intensity thresholds. The sum of the total area occupied by the cells is denoted by a_{cells} (C) The total area occupied by the dark pixels gives us the area of the colony a_{colony}. Colony packing fraction (ϕ_{colony}) is calculated as $\phi_{colony} = a_{cells}/a_{colony}$.

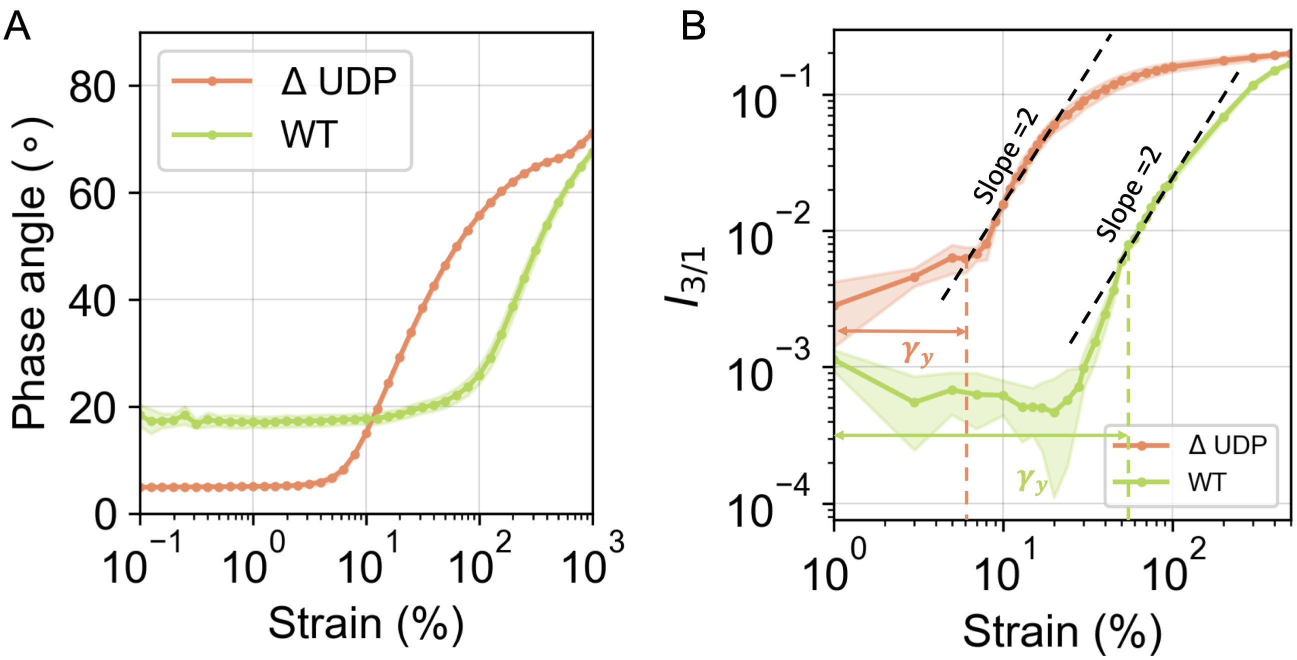

Figure S2: Figure shows the rheological measures obtained using oscillatory rheometry for the WT and ΔUDP biofilms. (A) Shows the variation in phase angle ($\delta°$) as a function of applied strain. (B) Shows the ratio of third harmonic to the first obtained using MITLaos. Lines of slope 2 are indicated in the figure. The yield strain (γ_y) corresponds to the minimum strain at which line of slope 2 starts and describes the onset of nonlinearity (irreversible deformation of structure) of the biofilms.

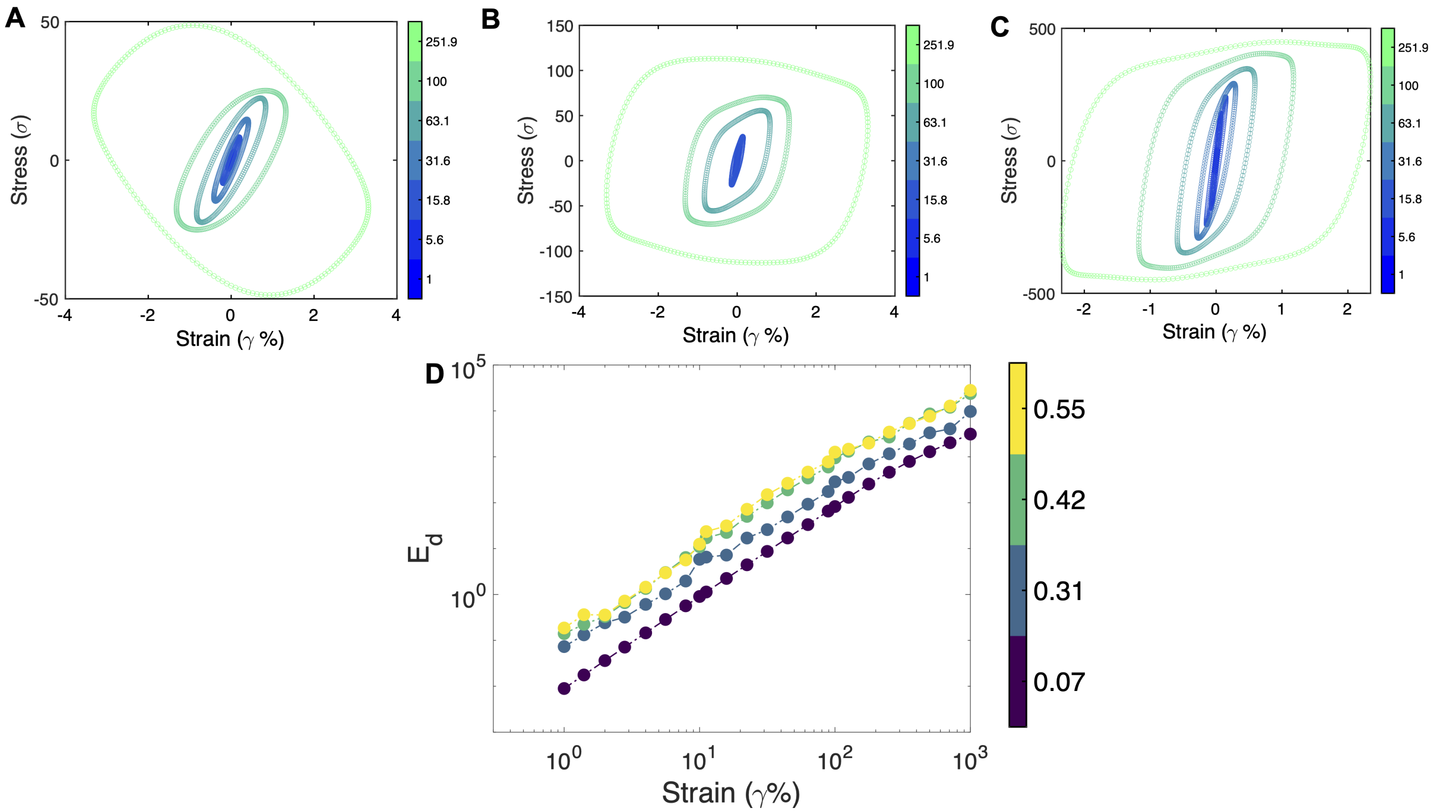

Figure S3: Elastic Lissajous Bowditch plots for mono-culture and co-cultured biofilms and energy dissipation characteristics (A) Elastic Lissajous Bowditch plots for $\phi = 0.07$ (B) Elastic Lissajous Bowditch plots for $\phi = 0.31$ (C) Elastic Lissajous Bowditch plots for $\phi = 0.55$ (E) Dissipated energy as a function of applied strain at 0.75 Hz for biofilms of varying packing fraction.

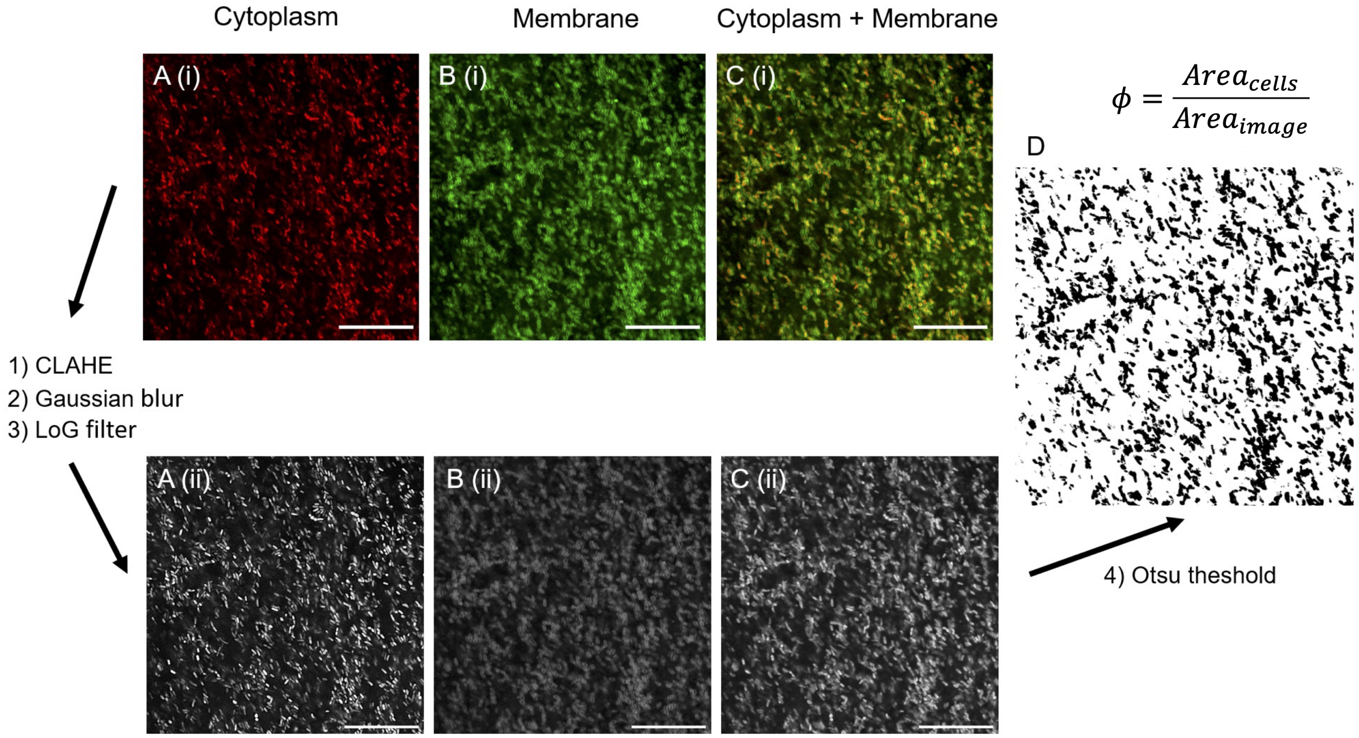

Figure S4: Figure shows the process of obtaining packing fraction (ϕ) from stained components of co-cultured biofilms of WT to ΔUDP in the ratio of 1:10. (A)(i) Shows the fluorescence emission from the cell cytoplasm labelled with Syto 63. (A)(ii) Shows a representative processed image slice of cell cytoplasm. The processing steps were as follows; Contrast-limited adaptive histogram equalization (CLAHE), Gaussian blur filter and a Laplacian of Gaussian filter. (B)(i) Shows the fluorescence emission from the membrane labelled with FM-464-FX. (B)(ii) Processed image slice of membrane fluorescence image, using the same steps as described in (A)(ii). (C)(i) The overlaid images from A(i) and B(i). (C)(ii) Processed image slice of the combined cell and membrane channels, using the same steps as described in (A)(ii). (D) After processing, the combined cell and membrane channel image was thresholded using Otsu's method. The packing fraction of the biofilm was then calculated as the area occupied by the cell/membrane divided by the total area of the confocal image. All scale bars are 20 μm.

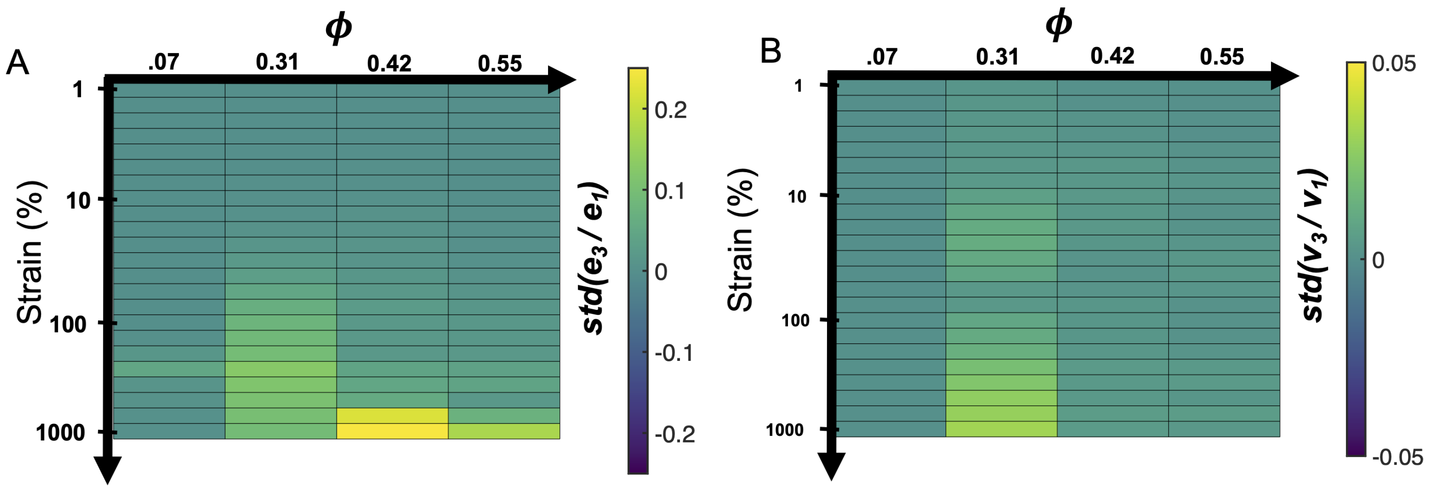

Figure S5: Standard deviation heatmaps (A) Heatmap represents one standard deviation of mean of e_3/e_1 (ratio of elastic Chebyshev coefficients) (B) Heatmap represents one standard deviation of mean of ν_3/ν_1 (ratio of viscous Chebyshev coefficients)

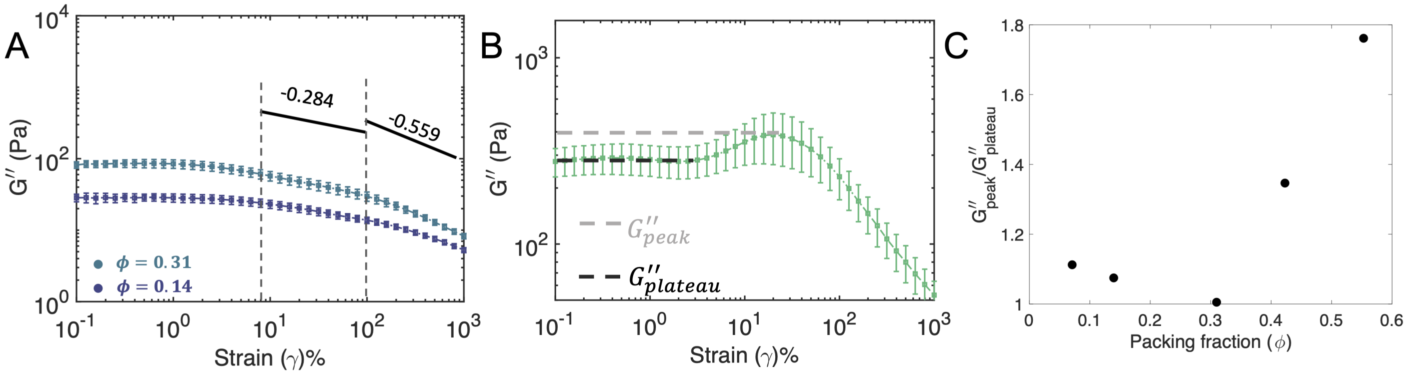

Figure S6: Additional rheological features of mono or co-cultured biofilms (A) Shows the plot of viscous modulus as a function of applied strain for intermediate packing fractions $0.14 \leq \phi \leq 0.31$. Line with slope of -0.284 is seen for $8\% \leq \gamma \leq 100\%$ and a decay rate of -0.559 is observed for $100\% < \gamma \leq 1000\%$ (B) Shows the variation in viscous modulus as a function of applied strain for $\phi = 0.42$. Peak and plateau modulus are indicated within the plot. (C) Shows a plot of ratio of peak to plateau viscous modulus as a function of packing fraction (ϕ).

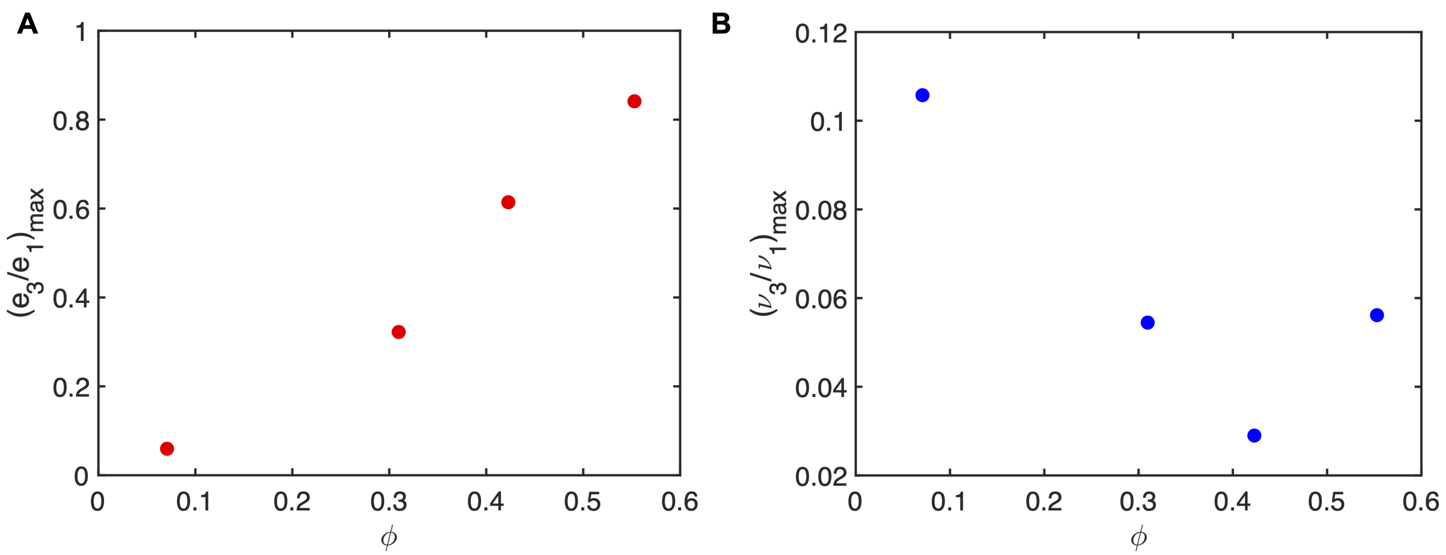

Figure S7: Variation of ratio of intra-cycle elastic or viscous Chebyshev coefficients as a function of packing fraction of biofilm (A) Plot of maximum ratio of Chebyshev elastic coefficients vs. packing fraction (ϕ). (B) Plot of maximum ratio of Chebyshev viscous coefficients vs. packing fraction (ϕ).

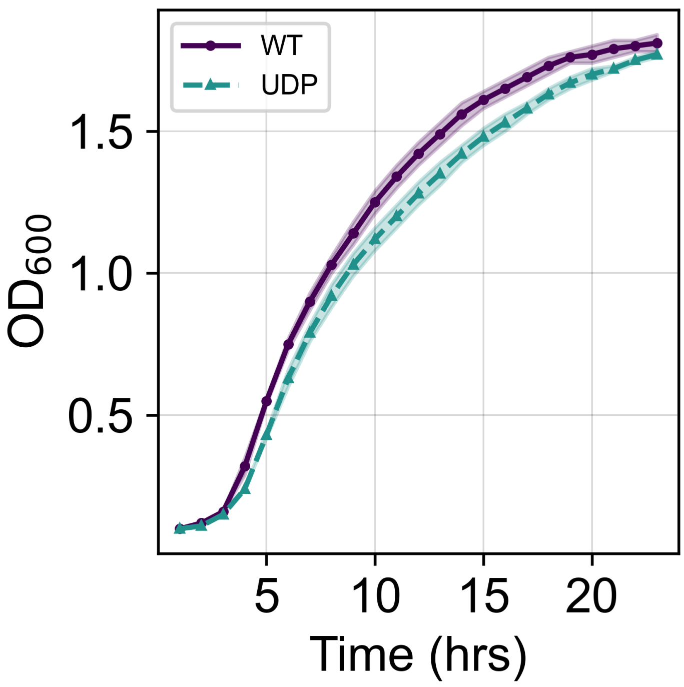

Figure S8: Growth curves of WT and ΔUDP strains in SOBG medium at 25°C. Error bars represent one standard deviation of mean (n=5).

ECM component	Run1	Run2	Run3	Run4	Mean±s.d.
Sugars (nmol/μL)	17.47	17.62	21.03	19.91	19.01 ± 1.75
Total protein ($\mu g/\mu$L)	28.6	24.9	24.6	21.9	25.00 ± 2.75

Table S1: Amounts of the sugars and protein extracted from WT

ECM component	Run1	Run2	Run3	Run4	Mean±s.d.
Sugars (nmol/μL)	1.98	3.61	3.39	3.28	3.06 ± 0.73
Total protein ($\mu g/\mu$L)	13.2	19.9	20.3	23	19.1 ± 4.16

Table S2: Amounts of the sugars and protein extracted from ΔUDP
